# The Diesel Exhaust in Miners Study provides no evidence for an increase in risk for lung cancer in miners exposed to diesel engine emissions

**DOI:** 10.1007/s10654-018-0455-z

**Published:** 2018-10-31

**Authors:** Matthias Möhner

**Affiliations:** Division of Work and Health, Federal Institute for Occupational Safety and Health, Nöldnerstr. 40/42, 10317 Berlin, Germany

**Keywords:** Diesel engine exhaust, Lung cancer, Risk estimation, Miners

## Abstract

The Diesel Exhaust in Miners Study is unquestionably the most suitable data material to date to examine a possible link between diesel engine emissions and lung cancer risk. But the results do not appear to be consistent in themselves. The crucial methodological problem in this study, however, has yet to be discovered, to which the lack of any description of age related information (year of birth, year of hire, year of first exposure, year of death) for the cohort as well as for the cases might have contributed. This information is important to understand the flaws in the analysis. It turns out that the year of birth is associated with the exposure, i.e. with the chance to be exposed over a certain period of time as well as with the chance to be an ever-smoker. A further important issue for the interpretation of the results is the validity of the data on smoking, which are mainly obtained from next of kin for decedents up to 50 years after death. Taking all these aspects into account, it can be concluded that only the SMR-analysis can be considered from all published results.

The results of the Diesel Exhaust in Miners Study (DEMS), which was based on a cohort and a nested case–control approach, were published in 2012 [1, 2]. DEMS is unquestionably the most suitable data material to date to examine a possible link between diesel engine emissions (DEE), measured as respirable elemental carbon (REC), and lung cancer risk. It can be assumed that the eight sub-cohorts of non-metal miners are sufficiently homogeneous in terms of social status and lifestyle and, in addition, that there is a strong contrast in exposure concentration between underground and surface activities in each of the sub-cohorts—a constellation rarely available in occupational epidemiology. The authors’ main results from the internal analysis of the cohort data as well as from the nested case–control study showed that the lung cancer risk in the highest exposure categories is up to five times as high as in the corresponding reference categories. The authors adjusted for location of employment as a binary variable (ever underground vs. surface only) in both models. In the case–control approach they even used the cross classification of location of employment, smoking status, and smoking intensity for adjustment. In contrast, the external comparison by standardized mortality ratios (SMRs) showed a slightly lower risk of lung cancer among ever-underground workers compared to surface-only employees.

Given its outstanding data base, the DEMS became the key study for the reclassification of DEE from group 2A (probably carcinogenic to humans) to group 1 (carcinogenic to humans) by the International Agency for Research on Cancer in 2012 [3]. The study results were subsequently evaluated by the Diesel Epidemiology Panel of the Health Effects Institute, which confirmed their suitability for a quantitative risk assessment [4]. Because the results appear not to be consistent in themselves, there have been a number of critical comments and re-analyses of the DEMS, most of which, however, have been focused on exposure assessment and adjustment for other occupational exposures like radon [5–16]. The crucial methodological problem in this study, however, has yet to be discovered, to which the lack of any description of age related information (year of birth, year of hire, year of first exposure, year of death) for the cohort as well as for the cases might have contributed. This information is important to understand the flaws in the analysis. The DEMS is therefore an impressive example of the growing rift between epidemiologists and their data, described recently in an essay by Rothman [17].

The cohort has some of the following characteristics, which are important for the analysis of the data and for the interpretation of the available results.

First, according to the definition of the cohort and the follow-up period from the year of dieselization (range 1947–1967) to 1997, the year of birth of all cohort members ranges from 1888 to 1978, assuming a retirement age of 60 years. Taking into account the age-specific mortality rates for lung cancer, it can be concluded that most lung cancer cases were born before 1920. The birth cohorts from 1940 and later are probably of no importance for the analysis.

Second, diesel technology was introduced in the mine virtually independently of the miner’s year of hire. Hence, the later the miner was born (assuming the same age at hire and the same length of employment), the longer was the maximum possible duration of underground employment under exposure to DEE. Consequently, the share of prevalent hires at the beginning of follow-up is of interest. It can be estimated for the cohort from the following equation, assuming a uniform fluctuation in the mines i.e. an equal annual number of incident hires in the period from the year of dieselization to 1 year before the end of follow-up:

$$p_{i} \times yd_{i} + \left( {1 - p_{i} } \right) \times \frac{{yd_{i} + 1996}}{2} = myfe_{i} ,$$

where *p*_*i*_ is the share of prevalent hires in the mine *i*, *yd*_*i*_ and *myfe*_*i*_ are the corresponding year of dieselization and mean year of first exposure to DEE, respectively. The estimate of *p*_*i*_ is then given by

$$p_{i} = \frac{{yd_{i} + 1996 - 2 \times myfe_{i} }}{{1996 - yd_{i} }}.$$

The weighted average of the mine-specific estimates yields the estimate for the whole cohort, which is about 25% based on the available data from the cohort study [1] (Table 1). The share of prevalent hires among lung cancer cases is undoubtedly much higher, probably at least twice as high.

**Table 1:** Estimated share of prevalent hires at study entry (year of dieselization) by study facility

Facility	Year of dieselization^a^	Mean year of first exposure to DEE^a^	Number of miners in the cohort^a^	Share of prevalent hires (%)^b^
A	1947	1967	1676	18.4
B	1964	1976	899	25.0
D	1950	1967	2105	26.1
J	1952	1969	1567	22.7
E	1959	1974	547	18.9
G	1962	1975	1135	23.5
H	1967	1975	1935	44.8
I	1956	1973	2451	15.0
All	1947–1967	1971	12,315	24.7

^a^Data from Table 1 in Attfield et al. [1]

^b^Calculated on the assumption of uniform fluctuation in all facilities over the study period

Third, smoking habits have also changed considerably over the birth cohorts. The maximum prevalence of smoking (which can be interpreted as the prevalence of ever smoking) in U.S. males increases from 55% for men born in 1890 to a plateau of about 76% for the 1915–1920 birth cohorts and then decreases continuously to 34% for those born in 1970 [18].

Thus, the year of birth is associated with the exposure, i.e. with the chance to be exposed over a certain period as well as with the chance to be an ever-smoker. Fortunately, however, the year of birth was used as a matching variable and thus cases and controls have comparable conditions in all of the 198 risk sets, even if a closer matching (the closest in terms of date of birth or at least within 1 year) would have been more favorable. An adjustment for smoking, as it is usually done by adding smoking as a categorical variable into the regression model, would have therefore been sufficient.

The authors justify their approach for adjustment with assumed negative confounding by smoking, which they are convinced results from the inverse relationship between smoking and DEE exposure among underground workers. However, the apparent link between smoking and DEE is induced by the link of both variables with the year of birth as described above. Moreover, the authors’ actual adjustment is based on the cross classification of location of employment and smoking. But location of employment is without a doubt highly correlated with the REC exposure concentration. Therefore, when comparing the risk of lung cancer between the categories of REC exposure, no adjustments should be made according to the location of employment. To further illustrate this, it is sufficient to look at Table 6 from the case–control results [2]. The upper tertile comprises miners with cumulative REC exposure ≥ 304 µg/m^3^-years. But a surface worker with an exposure concentration of 1.7 µg/m^3^ (the mean value over all mines, Table 2 [1]) has to work 178 years to achieve this value (or 95 years based on the highest mine specific mean value). As a result, virtually all miners in the upper tertile will belong to ever-underground workers.

A further important issue for the interpretation of the results is the validity of the data on smoking. It is well known that the accuracy of smoking data obtained from next of kin for decedents is questionable [19, 20]. In the present study the maximum of the time gap between year of death and year of data ascertainment for a lung cancer case can be as much as 50 years. As reported by the authors, 85% of the case subjects had died more than 10 years before data ascertainment [2]. In contrast, 40% of the controls were still alive at time of data ascertainment. Moreover, the contrast between the availability of information on smoking and history of respiratory disease raises further doubts about the validity of smoking data, especially for cases [12]. The smoking data for controls are therefore assumed to be considerably more valid than those for lung cancer cases. And they better reflect the general smoking habits in the exposure categories. This statement is also supported by data from Table 6 from the case–control results [2]. A comparison of smoking habits in controls (Table 6 [2]) shows that subjects in the 3rd tertile of cumulative REC lagged 15 years smoke more than subjects in the 2nd tertile, resulting in OR 2.09 [95% CI 1.07–4.09] for the comparison of heavy versus never smokers. Moreover, in contrast to the two lower tertiles, there is no significant difference in the 3rd tertile in terms of smoking habits between cases and controls. The calculation of ORs from simple 4 × 4-tables (excluding cases with unknown smoking status) yields an OR 2.57 for the comparison ever versus never smoker in the 3rd tertile, which is an extremely low risk compared to other studies. The corresponding ORs for the 1st and 2nd tertiles were 8.09 and 6.72, respectively. In contrast, the estimate from a study with very detailed smoking data gathered from the patients themselves yielded OR 13.4 [21]. These results reinforce the suspicion that the lack of validity of the smoking data has led to these conspicuous risk estimators. To account for data validity, it could be useful to make an additional adjustment for parameters like source of information on smoking or the difference between year of death and year of data ascertainment. So it turns out that the actual results of the case–control study are in no way reliable.

The discrepancy between the results of the external and internal analysis of cohort data is also likely to be explained by the year of birth as the link between cumulative exposure and smoking. Most lung cancer cases will have been born between 1910 and 1920, the birth cohorts with the highest likelihood of being allocated into the highest exposure group and the highest likelihood of dying of lung cancer by 1997. The internal analysis is based on a Cox model with age as the underlying time axis. To adjust for the birth cohort effect, a variable should be included in the Cox model describing the nonlinear change of ever-smoking prevalence by birth cohort in the reference population.

In summary, it can be concluded that only the SMR-analysis can be considered from all published results. The SMR for ever-underground workers was lower than for surface-only workers (SMR = 1.21 and 1.33, respectively)[1]. Of course, this analysis does not take smoking into account. But the case–control study has shown that ever-underground workers smoke more than surface-only workers. Moreover, these two groups are more homogeneous with respect to the distribution of birth cohorts than any categorization of miners by exposure parameters. The same seems to be true with respect to validity of smoking parameters. Hence, it can be assumed that a proper adjustment for smoking does not lead to a reversal of the risk ratio between ever-underground and surface-only workers. A rough estimate based on the published data supports this statement [22]. Consequently, the DEMS results provide no evidence that DEE could be associated with an increased lung cancer risk. The same applies to the reanalyses [7, 8], in which the virtually same models for the conditional logistic regression were used as in the original DEMS analysis. Although the alternative exposure estimates differ significantly from the DEMS researchers’ estimates, the enormous difference between surface and underground jobs remains.

Because the DEMS currently provides the world’s most comprehensive data for the analysis of lung cancer risk due to DEE, a reanalysis with an adequate adjustment for smoking would be useful. In addition to the original exposure estimates, the proposed alternative estimates [7] should also be considered along with a sub-division of the cohort according to location of employment at study entry. While the exposure contrast between the two groups is lower in such a sub-division, the groups are more comparable in terms of potential healthy-worker effects [11].

Finally, a comparison of the revised DEMS results with those of the study in German potash mining [23] would be of interest for the assessment of the lung cancer risk by DEE.
